# Supplementary material for: Safety Evaluation, Biogenic Amine Formation, and Enzymatic Activity Profiles of Autochthonous Enterocin-Producing Greek Cheese Isolates of the Enterococcus faecium/durans Group
Source: Microorganisms. 2021 Apr 8;9(4):777. doi: 10.3390/microorganisms9040777 (PMC8068099; doi:10.3390/microorganisms9040777)
Supplement: Supplementary file 1 [file microorganisms-09-00777-s001.zip › Microorganisms-1158123-supplementary/Microorganisms-1158123-supplementary.pdf]

SUPPLEMENTAL MATERIAL

Figure Legends

Figure S1

A) Agarose gel electrophoresis of multiplex PCR products of *van*A/*van*B in the clinical strain 315VR used as positive control. M: λDNA/*Hind*III (HT Biotechnologies); 1: *van*A; 2: *van*A/*van*B, 3: ddH_2O as negative control.

*van*A expected product at 545 bp, *van*B expected product at 368 bp.

B) Agarose gel electrophoresis of multiplex PCR products of *van*A/*van*B in the studied control LAB and the *E. faecium* and *E. durans* strains. M: λDNA/*Hind*III (HT Biotechnologies).

Figure S2

A) Agarose gel electrophoresis of PCR products for virulence genes *gel*E and *ace* in *E. faecalis* ATCC® 29212™, reference strain. M: λDNA/*Hind*III (HT Biotechnologies).

Agarose gel electrophoresis of PCR products for virulence genes: **B)** *gel*E and **C)** *ace* in the studied control LAB and the *E. faecium* and *E. durans* strains. M: λDNA/*Hind*III (HT Biotechnologies).

Figure S3

Agarose gel electrophoresis of PCR products for virulence genes: **A)** *IS*16, **B)** *hyl*, **C)** *agg* and **D)** *esp*A in the studied control LAB and the *E. faecium* and *E. durans* strains. M: λDNA/*Hind*III (HT Biotechnologies).
