# Supplementary figures and images for: Safety Evaluation, Biogenic Amine Formation, and Enzymatic Activity Profiles of Autochthonous Enterocin-Producing Greek Cheese Isolates of the Enterococcus faecium/durans Group
Source: Microorganisms. 2021 Apr 8;9(4):777. doi: 10.3390/microorganisms9040777 (PMC8068099; doi:10.3390/microorganisms9040777)

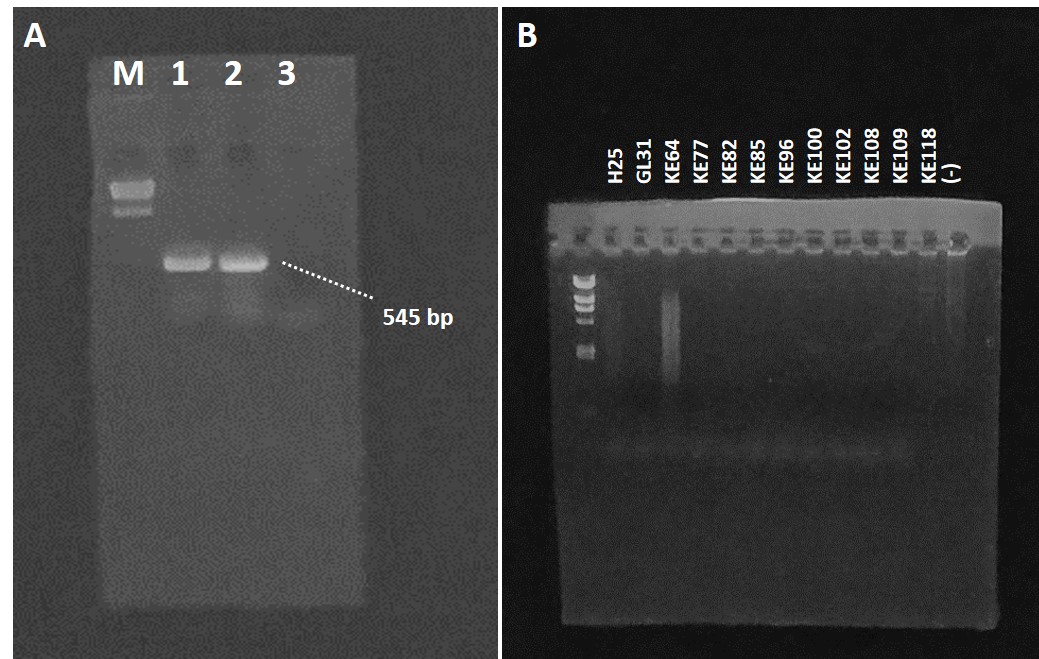

Supplement: Supplementary file 1 [file microorganisms-09-00777-s001.zip › Microorganisms-1158123-supplementary/Microorganisms-1158123_Suppl. Figure S1.jpg]

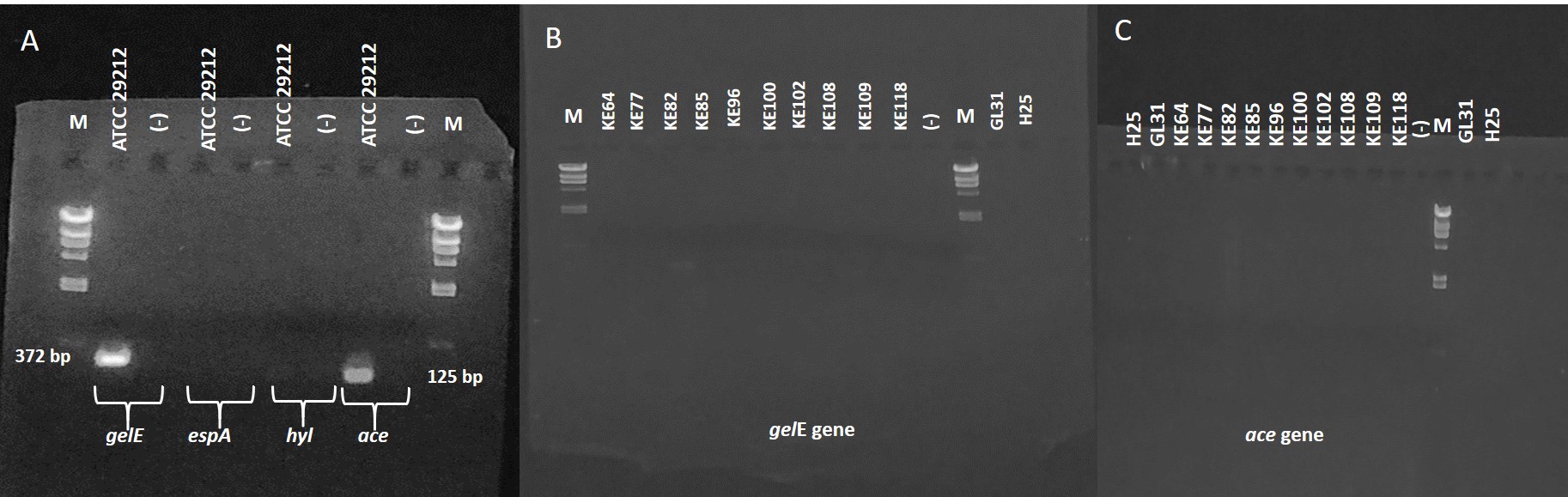

Supplement: Supplementary file 1 [file microorganisms-09-00777-s001.zip › Microorganisms-1158123-supplementary/Microorganisms-1158123_Suppl. Figure S2.jpg]

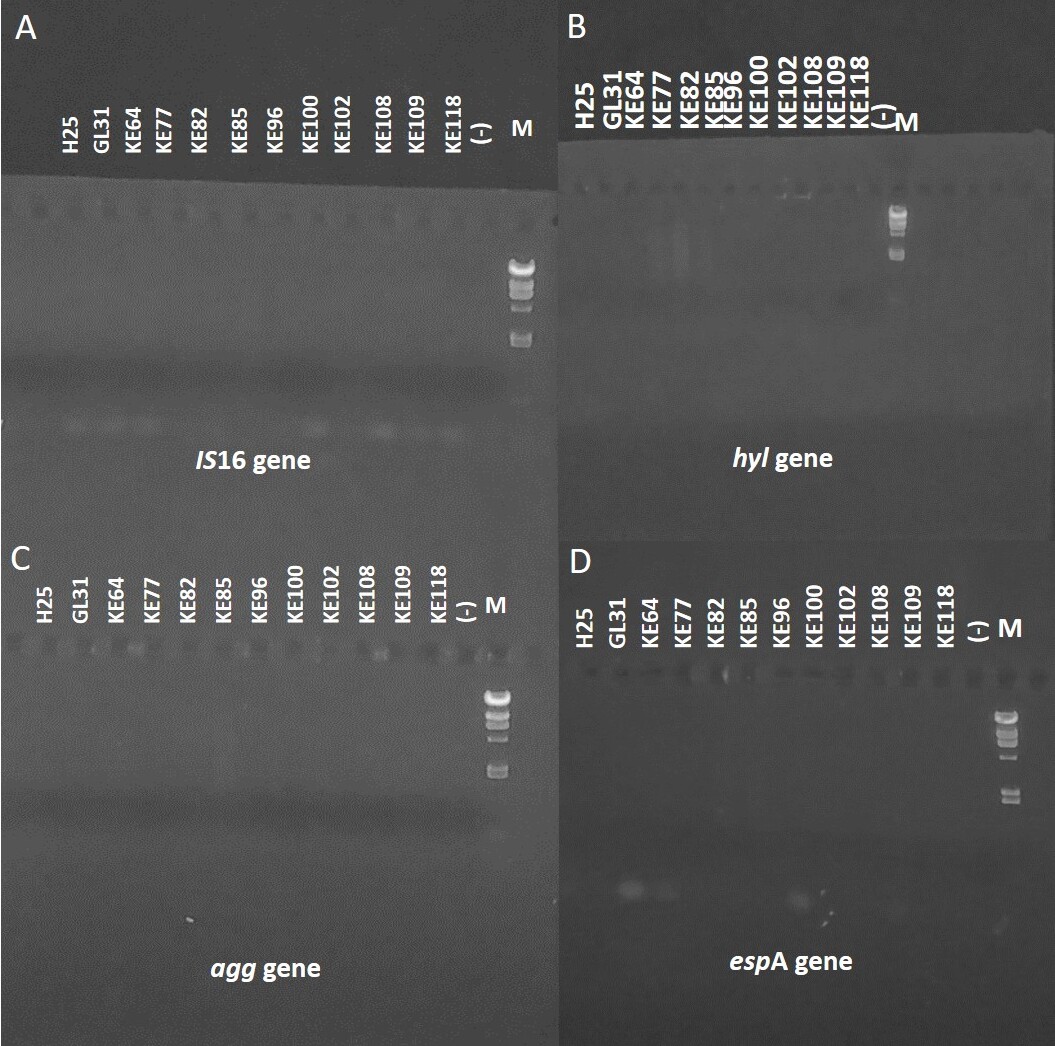

Supplement: Supplementary file 1 [file microorganisms-09-00777-s001.zip › Microorganisms-1158123-supplementary/Microorganisms-1158123_Suppl. Figure S3.jpg]
